# Clinical study to estimate correlation between graft thickness as measured by anterior segment Optical Coherence Tomography and visual recovery after manual Descemet stripping endothelial keratoplasty

**DOI:** 10.22336/rjo.2022.24

**Published:** 2022

**Authors:** Rakesh Shetty, Jaya Kaushik, Arun Kumar, Ankita Singh

**Affiliations:** * Department of Ophthalmology, Armed Forces Medical College, Pune, Maharashtra, India

**Keywords:** thin DSEK graft, pseudophakic bullous keratopathy, DSEK, corneal endothelial decompensation, ultrathin DSEK

## Abstract

**Purpose:** To estimate the correlation between graft thickness as measured by anterior segment Optical Coherence Tomography and visual recovery after manual Descemet stripping endothelial keratoplasty.

**Design:** Prospective observational study.

**Materials and methods:** This prospective observational study included 25 patients with pseudophakic bullous keratopathy, who underwent DSEK. Visual acuity in LogMAR units and estimation of graft thickness measured by Anterior Segment Optical Coherence Tomography (AS-OCT) was carried out on postoperative day 1, 3rd month and 6th month. Correlation analysis was performed between graft thickness and postoperative visual acuity on postoperative day 1, 3rd month and 6th month.

**Results:** The mean LogMAR visual acuity improved in all patients post DSEK - 1.47 (±0.48) to 0.75 (±0.38) LogMAR units. The average postoperative day 1 central corneal thickness was 724.96 µm (±77.59 µm), which decreased to mean central corneal thickness of 655.56 (±61.08 µm) on 3rd month and 633.48 (58.72) by the 6th month. Mean donor graft thickness on postoperative day 1, 3rd month and 6th month in this study was found to be 169.3 µm (±36.6 µm), 135.9 µm (±29.8 µm) and 127.5 µm (±31 µm) corresponding to a BCVA of 1.94, 0.86 and 0.75 LogMAR units respectively. On correlation analysis, thinner grafts were found to be associated with better postoperative BCVA especially on the 3rd and 6th month follow-up period.

**Conclusion:** This study found that a higher proportion of thinner grafts achieved better postoperative visual rehabilitation and earlier stabilization of visual acuity.

## Introduction

Endothelial keratoplasty has enabled great strides to be made in the visual rehabilitation of those afflicted with blindness due to corneal impairment, especially due to etiologies causing corneal endothelial dysfunction, such as Fuch’s Dystrophy, Congenital Hereditary Endothelial Dysfunction, Pseudophakic Bullous Keratopathy etc. [**1**-**4**]. It is an established fact that Descemet’s Stripping Endothelial Keratoplasty (DSEK) and Descemet Membrane Endothelial Keratoplasty (DMEK) are currently the preferred surgical procedures in comparison to Penetrating Keratoplasty for ocular indications involving endothelial dysfunction [**5**-**8**]. In spite of the remarkable progress made in the operation technique and instrumentation of DSEK [**1**-**3**,**9**], eyes undergoing DSEK seldom achieve visual acuity better than 6/ 12, clearly signifying that certain factors other than surgical technique may be responsible for the inability to achieve complete and optimal visual recovery following DSEK [**10**]. Although, better visual recovery is obtained, DMEK is still technically demanding and accompanied by high tissue wastage rates, which, in a resource limited setting, translates to lesser availability of the facility to the patient population [**11**,**12**]. This leaves a chiasm for a preferred surgical procedure that can combine the benefits of both DSEK and DMEK while negating their disadvantages. The studies have shown that DSEK performed using thinner grafts (preferably <100 µm) have faster visual recovery rates comparable with DMEK [**13**-**18**]. At the same time, many studies have also proven that graft thickness has no bearing on the final refractive state of the recipient eye post-surgery. In our study, a correlation between graft thickness as measured by Anterior Segment Optical Coherence Tomography (AS-OCT) and visual recovery post manual DSEK primarily on 3rd month and 6th month has been derived to determine whether thinner grafts are associated with faster visual recovery. It has been observed that thinner grafts cause lesser irregularities and consequently lesser posterior corneal higher order aberrations, leading to faster and better visual recovery.

## Material and methods

This prospective observational study was conducted at a tertiary hospital and included 25 patients with pseudophakic bullous keratopathy undergoing DSEK. Visual acuity in LogMAR units and estimation of graft thickness using AS-OCT was performed on postoperative day 1, 3rd and 6th month measured by AS-OCT. Statistical analysis was done to observe the correlation between graft thickness and postoperative visual acuity on postoperative day 1, 3rd month and 6th month. All cases underwent thorough pre and post operative ophthalmic clinical examination including slit lamp biomicroscopy, Snellen Visual acuity, direct, indirect ophthalmoscopy, and Anterior Segment Ocular Coherence Tomography (AS-OCT) [Carl Zeiss Meditech] to measure graft thickness on postoperative day 1, at 3 months and 6 months. The study included patients with uncomplicated Pseudophakic Bullous Keratopathy (PBK) and age above 18 years. Patients with previous corneal transplant, post op follow-up duration lesser than 6 months and co-existing vision affecting disorders (except for cataract) were excluded.

*Surgical technique*

The surgeries were performed by a single surgeon having a vast experience in Endothelial Keratoplasty procedures. The manual dissection of donor lenticule was carried out on an artificial anterior chamber before the surgery. The anterior lamellar dissection was done up to pre-Descemet level. An 8 mm trephine was used to cut the adequately sized donor corneal button and subsequently the endothelial side was marked. Thereafter, the lenticule was placed in Cornesol preservative media. After making a side port entry, the recipient bed was marked with an 8 mm trephine followed by Descemet membrane scoring, which was performed using a reverse Sinskey’s hook. A 5 mm superior limbal main entry incision was made to introduce the sheet glide into the AC through the main entry port. The lenticule was placed over the sheet glide with Healon GV to protect endothelium and inserted using a bent 26 G needle (cystitome) into the anterior chamber. The sheet glide was then removed. Air tamponade was maintained to ensure that the donor lenticule apposed well to host stroma. The incision was closed using 10-0 monofilament nylon sutures. Strict supine position was observed for the first 24 h by the patients. Postoperatively, patients were managed with tapering doses of topical steroids (Prednisolone 1%), topical antibiotics (Moxifloxacin 0.5%), and topical lubrication (Hydroxypropyl-methyl cellulose 0.3%). 

Central donor lenticule thickness on day 1, 3rd month and 6th month postoperatively was measured using AS-OCT. Thickness measurements were obtained centrally and manually through the visual axis. All measurements were obtained by a single operator as an average of 3 measurements.

*Statistical analysis*

Statistical analysis of the data was performed using SPSS (Statistical Package for social sciences) Version 20:0. Qualitative data variables were expressed by using Frequency and Percentage (%). Quantitative data variables were expressed by using Mean and SD. Correlation between DSEK lenticule thickness and BCVA (LogMAR) after the surgery on day 1, 3rd and 6th month was ascertained using Paired t-test. A p-value less than 0.05 was considered statistically significant.

## Results

This institutional prospective observational study, initially enrolled 28 eyes of 28 patients with Pseudophakic Bullous Keratopathy for a period of 6 months follow up. However, among these patients, one patient was lost to follow-up, one eye had optic nerve dysfunction, which was diagnosed after the procedure, while one eye had prolonged cystoid macular edema, probably as a result of the previous cataract surgery. After exclusion of these three eyes, the results from the remaining 25 eyes were studied. The demographic characteristics of the study population are shown in **Table 1**. Age distribution of the study population was the one depicted in **Table 2**.

**Table 1:** Characteristics of study population

Characteristics		Data
No. of patients		25 (25 eyes)
Age	>50 years	18
	<50 years	07
Sex	Male	16
	Female	09
Laterality	Left	13
	Right	12
Mean preoperative BCVA		1.47 (±0.48) LogMAR units

**Table 2:** Age distribution of study population

Age group (years)	Number of patients	Percentage (%)
≤40	3	12.0
41-50	4	16.0
51-60	2	8.0
61-70	10	40.0
>70	6	24.0
Total	25	100.0

**Fig. 1:**
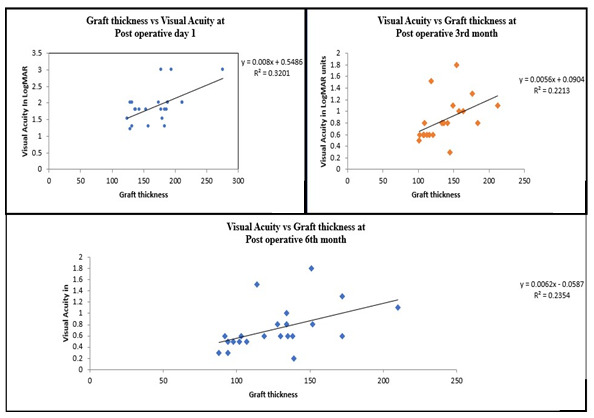
Relationship between visual acuity in LogMAR units and graft thickness on postoperative day 1, 3rd and 6th month

**Fig. 2:**
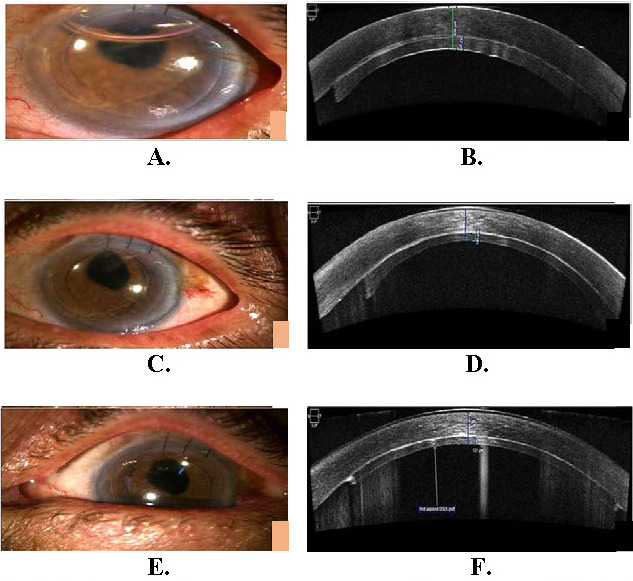
**A, B** Clinical picture and AS OCT post DSEK day 1; **C, D** Clinical picture and AS OCT post DSEK on the 3rd month; **E, F** Clinical picture and AS OCT on post DSEK 6th month

The mean pre-treatment visual acuity of the 25 eyes was LogMAR 1.47 (±0.48) units, which improved in all patients post DSEK - 1.47 (±0.48) to 0.75 (±0.38) LogMAR units. The postoperative day 1 mean central corneal thickness was 724.96 µm (±77.59 µm), which decreased to 655.56 (±61.08 µm) on 3rd month and 633.48 (58.72) by the 6th month. Donor lenticule thickness ranged from 98 to 212 µm (median = 134 µm). Mean donor graft thickness on postoperative day 1, 3rd month and 6th month in this study was found to be 169.3 µm (±36.6 µm), 135.9 µm (±29.8 µm) and 127.5 µm (±31 µm) corresponding to a BCVA of 1.94, 0.86 and 0.75 LogMAR units respectively. On correlation analysis (**Fig. 1**), thinner grafts were found to be associated better with postoperative BCVA, especially on the 3rd and 6th month follow-up period. A statistically significant, positive correlation was found between donor lenticule thickness and visual acuity in LogMAR units, indicating that thinner grafts were associated with better visual acuity, with a Pearson correlation coefficient of 0.470 (p<0.001 by paired t test) on the 3rd month of follow-up, which increased to 0.485 (p<0.001 by paired t test) on the 6th month of follow-up. Median graft thickness of all grafts was calculated on the 3rd month follow-up and was found to be 134 µm. 13 patients achieved stable visual acuity by the 3rd month itself (i.e., visual acuity on the 3rd month and 6th month was the same in the study). Of these, 69% of the eyes were those that received grafts <134 µm, thus suggesting faster recovery of visual acuity in patients with thinner grafts. Most patients achieved excellent visual gain as compared to their preoperative visual acuity with an average gain of 0.65 LogMAR units (p<0.001 by paired t test) at 6 months post-operatively. Overall, when all grafts were considered, the mean visual gain at 6 months in patients with grafts thickness was <134 µm (0.71 LogMAR units), though it was more than in those with thicker grafts (0.59 LogMAR units), not being statistically significant (p=0.289 by paired t test). As early as 3 months, a BCVA more than 6/ 18 was recorded in 2 out of the 25 patients. BCVA kept on increasing with time in 8 patients (32%), reaching more than 6/ 18 at 6 months. Postoperatively, in two cases, interface fluid was noted, which was managed successfully by the drainage of fluid using air injection. At the 6-month follow up, the mean donor lenticule thickness was 128 µm. In three cases, residual corneal haze was observed (all longer duration cases with history of >6 months of PBK). **Fig. 2 A-F** shows the clinical photographs and AS-OCT scan of a patient post DSEK on day 1, 3rd month and 6th month of follow-up.

## Discussion

Posterior Lamellar keratoplasty has come a long way since inception when it was first performed by Tillet in 1956 and then refined by Melles in 1998. Terry and Ousley introduced deep lamellar keratoplasty in the U.S. in 2000 and thereafter endothelial keratoplasty has continued to rise in popularity [**19**-**22**]. In 2005, 4% of all cornea transplants in the United States were represented by endothelial keratoplasty as compared to 44% in 2008. In the successive era, various methods of graft preparation, insertion and manipulation were deliberated upon leading to continued refinement of microsurgical technique of posterior lamellar keratoplasty. As per Eye Bank Association of America eye banking statistical report 2019, Descemet Stripping Automated Endothelial Keratoplasty (DSAEK) is at present the most widely employed procedure performed for endothelium transplantation in patients with Fuchs endothelial dystrophy or pseudophakic bullous keratopathy [**10**,**23**]. In India, PBK is second only to infective causes in the etiology of corneal blindness and DSEK continues to rise in popularity as the preferred treatment modality, as a favorable number of surgeons are acquiring surgical skills for same [**24**]. In DSEK, the host endothelium and Descemet’s membrane (DM) are replaced by the donor endothelium and DM, along with a small amount of posterior stromal thickness, through a corneal or corneoscleral incision [**3**,**4**,**11**]. Although DSEK is a successful procedure, visual outcomes following even uncomplicated cases remain sub-optimal despite minimal intra-op graft manipulations and clear post op graft [**25**-**29**]. Optical degradation especially increased optical aberrations and light scatter has been speculated as the reason for the same in many studies, factors implicated including, corneal edema, graft-host interface irregularities and posterior corneal surface thickness changes among others [**25**,**26**,**30**-**37**]. Additionally, a doubt about the role of graft thickness in the final visual recovery remains until date, which, if established, would be an easier factor to modify enabling better visual outcomes, especially in resources limited settings, wherein manually dissected DSEK is still practiced. Preparing thinner DSEK grafts is easier as compared to DMEK grafts, thus reducing tissue wastage while improving quality of transplant tissue. When compared with DMEK, thinner DSEK grafts have the advantage of lower graft dislocation rates of conventional DSEK and can be implanted even in aphakic eyes, those with extensive iris trauma or anterior chamber intraocular lens. Therefore, this study sought to highlight the correlation between graft thickness and rate of visual recovery post manually dissected DSEK graft implantation, in patients with Pseudophakic Bullous Keratopathy. While many studies, such as those performed by Nieuwendaal et al., Daoud et al. and Clynenbrugel et al. have found no correlation between visual results and postoperative graft thickness, studies performed by Busin et al., Neff et al. etc., strongly suggest that thinner DSAEK grafts were associated with faster visual recovery and better final post operative visual acuity [**3**,**14**-**17**,**34**,**38**-**41**]. The better visual outcomes following DMEK also support the theory of thinner grafts, being associated with better visual outcomes, although the complexity of the procedure, as well as the high graft detachment rate along with higher proportion of endothelial cell loss makes its use less popular, especially in resource limited settings [**12**,**42**-**45**]. This study successfully ascertained the positive correlation between graft thickness and visual acuity in LogMAR units, with thinner grafts achieving faster visual recovery as compared to thicker grafts overall. A higher proportion of grafts with thickness <134 µm (median thickness of grafts harvested in this study) stable visual acuity on the 3rd month itself, when compared to grafts with lenticule thickness >134 µm. Also, the mean visual gain was 0.71 LogMAR units on the 6th month in grafts thinner than 134 µm as compared 0.59 LogMAR units in the thicker graft group. These findings suggested that the rate of visual recovery was faster in patients in patients who received thinner grafts as compared to those with thicker grafts. An important relationship between graft thickness and final visual acuity may exist when grafts used are much thinner than those implanted in this study, as suggested by early results of DMEK, in which no donor stroma is transplanted, as well as of those of ultra-thin and nano-thin DSEK as brought out very recently. However, a potential disadvantage of using thinner grafts is the relative difficulty in the unfolding of graft as compared to thicker grafts. In addition, no difficulty in unfolding of grafts was encountered in this study. No significant difference in visual acuity was found between patients younger than 50 years, as well as those above 50 years, suggesting that age may not be a factor in the visual rehabilitation in uncomplicated cases. 

Furthermore, most studies have documented continued improvement in visual acuity in DSEK patients especially at 1 year and 2 years follow-up period after the surgery and a longer-term study would have possibly brought out a difference between thinner and thicker grafts with respect to stable visual acuity finally attained by the patients.

Thus, as thinner grafts are associated with faster and better visual recovery and as these are technically easier to prepare compared to DMEK grafts, thinner DSEK grafts should be formed, especially in resources limited setups, to provide patients with benefits of both DSEK and DMEK while avoiding their disadvantage.

Among the limitations of this study, the small sample size would be one. A study with a larger sample size would have allowed a better comparison of surgical results. As most studies documented the continued improvement of vision at 1 year and 2 years post DSEK surgery, a longer duration study would have been able to quantify the difference in visual gain based of thickness of corneal graft, as well as visual recovery rates. In addition, to minimize the measurement error, the mean of 3 thickness measurements taken by a single operator has been used, the measurement precision of AS-OCT (in the order of 10-20 µm) becomes more important as thinner structures are measured and could constitute a source of error in this study. 

## Conclusion

Endothelial keratoplasty has made great progress and seen many improvements in microsurgical techniques over the years. With the advent of DSEK for treatment of corneal endothelial dysfunction related corneal blindness, visual recovery rates became better, faster, and presented lower complication rates especially as compared to penetrating keratoplasty. Although numerous studies have been conducted with conflicting findings regarding the correlation of graft thickness and post DSEK visual rehabilitation, the recent trends towards thinner grafts, as well as documented success of ultra-thin and nano-thin DSEK points towards beneficial effect of forming thinner DSEK grafts, have been observed. This study found that a higher proportion of thinner grafts achieved better post-operative visual rehabilitation and earlier stabilization of visual acuity. However, given the limitation of the small sample size and short duration of study, larger sample size multi centric studies with longer duration are required to conclusively elucidate the role of thin DSEK grafts in the visual recovery of patients. 

**Conflict of Interest Statement**

The authors state no conflict of interest. 

**Informed Consent and Human and Animal Rights statement**

Informed consent has been obtained from all the patients included in the study.

**Authorization for the use of human subjects**

Ethical approval: The research related to human use complies with all the relevant national regulations, institutional policies, it is in accordance with the tenets of the Helsinki Declaration and has been approved by the review board of Armed Forces Medical College, Pune, Maharashtra, India.

**Acknowledgements**

None. 

**Sources of Funding**

None. 

**Disclosures**

None. 
